# An Exploratory Study on Farming System and Meat Quality of Black Alpine Pig

**DOI:** 10.3390/ani16010022

**Published:** 2025-12-21

**Authors:** Annalaura Lopez, Federica Bellagamba, Raffaella Rossi, Margherita Greco, Edda Mainardi, Carlo Corino, Vittorio Maria Moretti

**Affiliations:** Department of Veterinary Medicine and Animal Science, University of Milan, Via dell’Università 6, 26900 Lodi, Italy; federica.bellagamba@unimi.it (F.B.); raffaella.rossi@unimi.it (R.R.); margherita.greco@unimi.it (M.G.); edda.mainardi@unimi.it (E.M.); carlo.corino@unimi.it (C.C.); vittorio.moretti@unimi.it (V.M.M.)

**Keywords:** autochthonous pig, biodiversity, mountain areas, meat quality

## Abstract

This study aimed to provide the first characterization of the traditional farming systems and meat quality traits of the Black Alpine pig (BAP), an ecotype under recovery originating in the Italian Central Alps, reared under real, actual conditions. Productive and reproductive data were collected through on-farm visits from six BAP farms located in Northern Italy. Longissimus dorsi (LD) muscle and backfat samples from BAPs were collected in the same farms and analyzed for color, tenderness, proximate composition, and fatty acid profile. For exploratory purposes and to provide a descriptive benchmark of commercial pork (CP) quality, loin samples were purchased from the retail market. PLS-DA served as a discriminant model between BAP and CP meat samples on a multivariate data matrix. BAP farming was characterized as an *en plein air* system with high outdoor access and reliance on natural vegetation. BAPs showed high rusticity and slow growth. The LD muscle was characterized by a firm texture, an intense dark-red color, and a high proportion of polyunsaturated fatty acids, reflecting traditional rustic meat features associated with the interaction between dietary, environmental, and genetic factors. Our preliminary results provided novel insights into BAP farming practices and meat quality, supporting the maintenance of this genetic resource through traditional farming and the preservation of biodiversity in alpine livestock, offering exploratory evidence of the current BAP farming system.

## Simple Summary

This work describes the traditional way of raising the Black Alpine pig, a local mountain pig from Northern Italy, and examines the quality of its meat. Six small farms were monitored to record animal growth, reproduction, and farm conditions. Meat and fat samples were collected from these farms and compared with those from ordinary commercial pork. The Black Alpine pigs were raised mostly outdoors, feeding on grass, roots, and local plants, and showed strong adaptation to their mountain environment. Their meat was darker red, firmer, and contained more unsaturated fats than commercial pork. These characteristics reflect the influence of the animals’ diet, their genetics, and the natural environment in which they live. These results highlight how preserving traditional mountain farming not only supports animal welfare and local culture but also helps maintain biodiversity and produces meat with unique quality traits.

## 1. Introduction

In rural areas, farming of native pig breeds could represent a significant economic resource and an opportunity to stimulate diversified agriculture through an increase in the market and value of food products that meet actual societal concerns, like biodiversity preservation, quality, animal welfare, and sustainability [1]. Local breed populations are generally composed of a small number of animals and, according to the FAO, animal biodiversity is vital to efficient and sustainable food production in the world and to meeting the different needs of human societies [2].

The FAO lists several Italian native pig breeds, but only eight have been recognized in the Italian Pedigree Register (https://suis.anas.it/). Other native populations included in the FAO Domestic Animal Diversity Information System (https://www.fao.org/dad-is/en) have not obtained an official registration, and a very high number of populations have been declared extinct [3]. The management and preservation of genetic variability represent an advantage for breeders, both for maintenance of the breed and to avoid inbreeding. Moreover, to improve local breed sustainability, it is important to obtain scientific assessments of their productivity and product quality [4].

The Black Alpine pig (BAP) is an ecotype that originated from the consolidation of three relict pig populations historically located in Valtellina and South Tyrol. Currently, 55 farms are engaged in its breeding, including 29 situated in the mountainous regions of Northern Italy, 12 in Switzerland, 11 in Austria, and 3 in the Bavarian Alps. In Italy, the estimated population comprises approximately 400 animals, of which 85 are recognized as breeding stock. These animals are distributed across several provinces, including Sondrio, Como, Aosta, Alessandria, Belluno, Trento, and Bolzano [5]. The BAP is a robust, pasture-adapted pig, well suited to Alpine environments. It reflects the traditional pig farming systems once widespread in the Alps, prior to the expansion of commercial hybrid pigs selected primarily for rapid growth and lean meat yield. Breeds like the BAP are particularly relevant for their resilience, being highly adapted to outdoor conditions and requiring minimal management. BAPs exhibit good temperament, high stress tolerance, high social behavior within groups, and a generally quiet attitude toward humans. Adult sows typically weigh between 130 and 160 kg, while boars reach 150 to 180 kg. Height at the withers ranges from 60 to 70 cm in sows and from 70 to 75 cm in boars.

Morphologically, the BAP shows a short, compact, and muscular body with a straight or slightly convex back. The shoulders are fine, while the thighs are long with straight buttocks. The tail is thin and elongated. The head is long with a straight fronto-nasal profile and large, forward-projecting ears. The coat color varies from black to golden or dark red and brown, with long bristles that may show reddish split tips along the dorsal line. The skin is pigmented on the back and outer limbs but pink on the abdomen and inner forearms and thighs. The limbs are long and robust, with strong feet and dark, open hooves. This morphological characterization was prepared in accordance with the descriptors and criteria outlined in the Italian guidelines of the Ministry of Agricultural, Food, and Forestry Policies [6].

**Figure 1:**
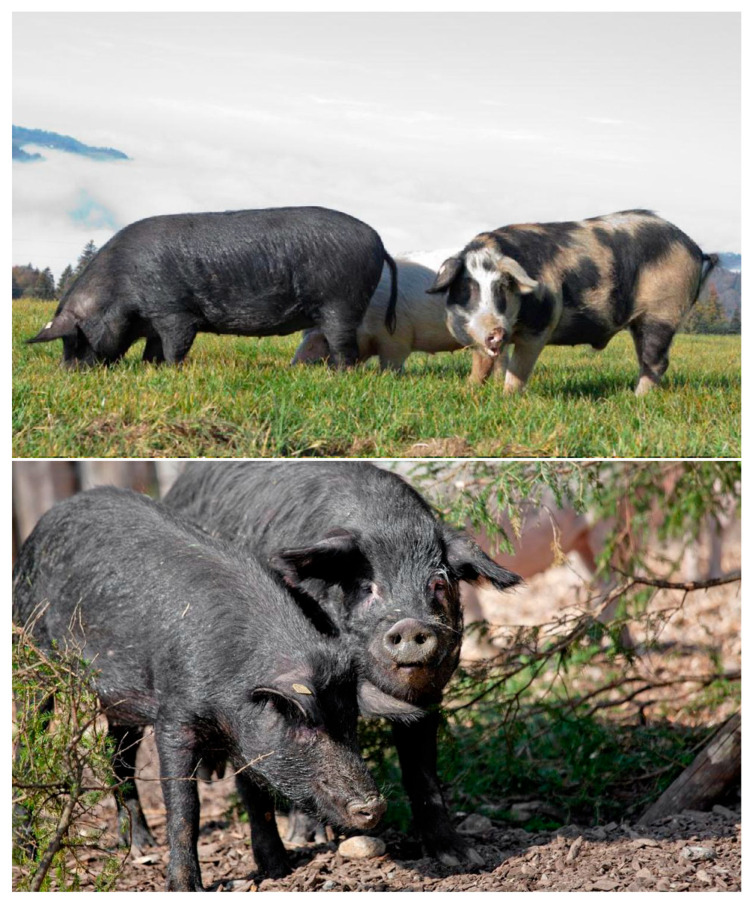
Specimens of Black Alpine pig with different coat colors in the alpine environment. Photo ©ProPatrimonioMontano (https://patrimont.org/en).

In Figure 1, some specimens of BAPs in their environment are shown.

Effectively, native breeds are mostly exploited in outdoor rearing systems, allowing for natural pig behavior, such as exploration and foraging, which have been demonstrated to produce higher-quality meat and products. In this context, a more detailed characterization of the meat quality traits of the BAP is essential to supporting greater market penetration and facilitating the recognition of high-quality, potentially label-certified products. Traditional production systems, as well as final products, have high economic value in the market because consumers recognize their quality, including environmental and social value and ethical and cultural impact [7,8,9,10].

Notably, the differences in meat quality between commercial hybrids and autochthonous pigs concern intramuscular fat content and fatty acid profiles, which affect sensory attributes and the meat transformation process. Meat from native pigs farmed in outdoor systems also has a higher proportion of functional compounds, such as n-3 polyunsaturated fatty acids, including linolenic acid, and α-tocopherol [9,11,12,13,14]. In extensive rearing, the continuous movement of the animals and the slower growth cause the meat to become more compact and the fat more well distributed, conferring an excellent organoleptic quality on the final product [10]. Similarly, a relationship has been evidenced between the extensive farming systems of native pig breeds and the profile of aromatic compounds detected in seasoned products. Szyndler-Nędza et al. [15] reported that the feeding regime (acorns vs. concentrate) influenced the fatty acid composition of meat from pigs of the native Złotnicka Spotted breed. Similarly, other studies have investigated the volatile compound profiles of seasoned lard, salami, and dry-cured ham from Sicilian Black [16,17] and Cinta Senese meat [18].

Despite growing interest in autochthonous breeds, the Black Alpine breed remains largely undocumented. The lack of information on its productive and reproductive performance, farming conditions, and meat quality profile creates a knowledge gap that limits the development of valorization strategies and opportunities for improving economic performance [19].

This study was designed to fill this knowledge gap by providing a comprehensive overview of the farming systems currently adopted for the Black Alpine Pig, with a focus on herd management, feeding and grazing strategies, and reproductive practices, and characterizes the meat quality traits of this autochthonous breed within the variability of its production context. The aims of this research are to provide evidence of the current phenotype of the BAP as a genetic resource to sustain alpine livestock farming and to provide a marketable meat associated with specific quality features.

This work has contributed to the registration of the Black Alpine Pig in the Italian Register of Agricultural and Food Biodiversity. This recognition confirms the uniqueness of this pig at both the scientific and national levels and will support its protection and valorization, promoting products derived from its rural supply chain.

## 2. Materials and Methods

### 2.1. Farm Management and Data Collection

**Figure 2:**
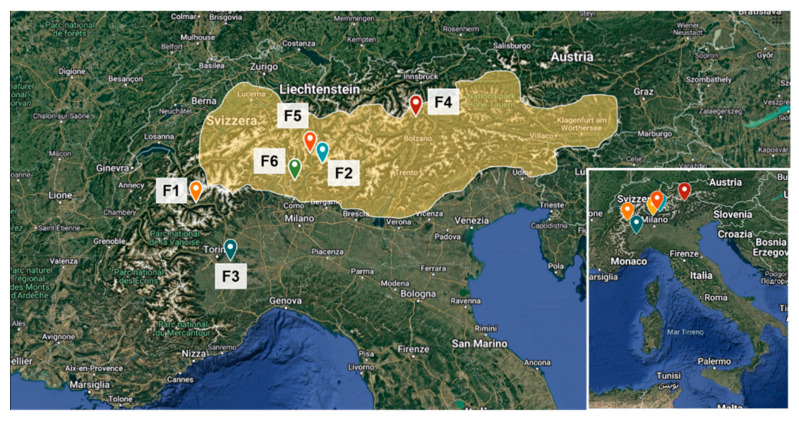
Map presenting the distribution of farms involved in the study (F1–F6). In yellow, the distribution across regions of Black Alpine pig is highlighted. The map was developed using the My Maps tool by Google.

Farms involved in this study, recruited based on farmer availability, were distributed over four Italian regions, including Valle d’Aosta, Piemonte, Lombardia, and Trentino-Alto Adige. The investigated farms varied considerably in size. The distribution and location of farms are presented in Table 1 and Figure 2.

All the data on farm management, breeding systems, and productive characteristics of animals in the various breeding groups were collected through a structured survey of the farmers and a direct interview during meat sampling. In addition, direct observations were made to verify the information provided by the farmers. Particular attention was paid to documenting traditional management practices, such as breeding strategies, feeding regimes, and housing conditions.

The data collection focused on (i) management practices: housing system, feeding strategies, environmental enrichment, and land use; (ii) farm structure and herd composition: total farm area, area allocated to pigs, and the number of animals by category (sows, gilts, boars, fattening pigs); (iii) morphological characteristics of pigs: body measurements and physical traits of the animals, including the weights and the heights of breeding stock; (iv) productive parameters: growth performance and slaughter data; (v) reproductive management and performance: breeding methods, farrowing management, and reproductive parameters.

### 2.2. Meat Sampling

Meat sampling was conducted between 2024 and 2025. The study included fifteen pigs, both males and females, with live weight at slaughter ranging from 140 to 195 kg and ages between 26 and 79 months, collected in the six farms. Due to the limited availability of the population, samples were collected exclusively during routine slaughter established by farmers. Meat samples from BAP were collected 24 h post-mortem. From each animal (n = 15), two samples of *Longissimus dorsi* (LD) muscle (2.5–3.0 cm thick) between the 10th and 14th ribs were sampled. Samples of backfat (BF) were also collected (n = 15) from the same anatomical location.

To provide a descriptive benchmark of commercial pork (CP) quality, LD muscle samples (n = 15) were purchased from the retail market. Due to the lack of production information regarding CP, these samples were included to offer only a contextual comparison with Black Alpine Pig meat in the multivariate analysis. The same anatomical portion as BAP was sampled for CP samples collected on the market.

All the samples were transported to the university laboratories under refrigeration and subsequently stored at −20 °C for the determination of physical and chemical parameters.

### 2.3. Color and Texture

After thawing for 24 h at 4 °C, the LD muscle samples were used for the determination of pH, color parameters, and texture measurements. pH measurements were conducted using a portable pH meter (HI98191; Hanna Instruments, Vila do Conde, Portugal) equipped with a meat-penetrating probe. All analyses were made in triplicate.

The color parameters lightness (L*), redness (a*), and yellowness (b*) were determined using a Chroma Meter CR-300 (Minolta Camera Co., Osaka, Japan). The device was standardized before use with a white calibration plate (CR-A43; Minolta Camera Co., Osaka, Japan). Measurements were taken with an 8 mm diameter measuring area under an illuminant C light source at a 0° viewing angle, with the spectral component included. Color values for each sample represent the average of four surface measurements taken at different positions. Color was measured after 30 min of meat blooming.

A fresh, 25 mm thick slice from each LD muscle sample was placed in a plastic bag and cooked to an internal temperature of 70 °C in a water bath. Internal temperature was monitored during cooking with a hand-held temperature probe. Cooked samples were allowed to cool for 30 min and then were vacuum-packed and stored at 4 °C for 24 h. Then, the LD muscles were prepared for textural analyses, removing the edges of the steak. Four strips were cut from each loin (15 mm width × 15 mm length × 15 mm height) and were used in a compression test via Texture Profile Analysis (TPA). From the same steak, four cylinders (12 mm diameter × 25 mm length) were cut using a puncher parallel to the longitudinal orientation of the muscle fibers for Warner–Bratzler Shear Force (WBSF) analysis [20].

Textural assessments were conducted using a TA-XT Plus Texture Analyzer (Stable Micro Systems, Godalming, UK), along with its accessories and software, for data acquisition and analysis. The TPA set consisted of a cylinder probe of 30 mm in diameter (P/30) and a flat platform. The parameters used in the compression test were set as follows: pre-test speed, test speed, and post-test speed = 3 mm s^−1^ target mode strain = 50%; time = 20 s; trigger type = force; trigger force = 10 g. The WBSF set consisted of a “V” slot blade according to the USDA Standard (HDP/WBV) and a slotted blade insert. The parameters used in the compression test were set as follows: compression mode; pre-test speed = 2.0 mm s^−1^; test speed = 2.0 mm s^−1^; post-test speed = 10.0 mm s^−1^; target mode distance = 20 mm, trigger type force = 10 g.

### 2.4. Proximate Composition and Fatty Acid Analysis

After thawing for 24 h at 4 °C, the LD muscle samples were minced using a blade homogenizer. Chemical composition analysis followed the protocols established by the Association of Official Analytical Chemists [21]. Duplicate measurements were performed for moisture determination (method 985.41), ash content (method 920.153), and protein quantification (method 928.08). Total lipids were extracted from ~2 g of minced sample using a mixture of chloroform and methanol (2:1, *v*/*v*) [22] and quantified gravimetrically in duplicate. After that, a pool of the two extracts from each sample containing 10 mg of lipids was recovered, dried under a gentle stream of nitrogen (37 °C), and used to prepare fatty acid methyl esters (FAMEs) before gas-chromatographic analysis. Briefly, the lipids were recovered in 1 mL toluene containing tricosanoic acid (C23:0) as an internal standard (200 μg mL^−1^), and FAMEs were prepared by adding 2 mL methanolic hydrogen chloride [23], keeping the sample in an oven (50 °C) overnight. The FAMEs were then extracted from the mixture using 2 mL hexane; the solution was cleaned using 2 mL K_2_CO_3_ 1 M and dried under a gentle stream of nitrogen (37 °C). All the FAMEs were finally recovered in 1 mL hexane and transferred into a 2 mL amber vial for gas-chromatography; 1 µL of the sample was injected.

Fatty acids were separated using a TRACE™ TR-FAME capillary column (Thermo Fisher Scientific, Waltham, MA, USA; 60 m × 0.25 mm, ID × 0.25 μm film thickness) under the following conditions: carrier gas—He, flow rate—1 mL min^−1^, injector temperature—250 °C, split mode (split ratio 50:1), FID temperature—250 °C. The oven temperature program was set as follows: 100 °C maintained for 1 min; 5 °C/min ramp up to 180 °C; 2.5 °C/min ramp up to 250 °C and maintained for 2 min (total run: 47 min). FAMEs were identified by comparing their retention times with those of various mixtures of analytical standards. These included a mix of 37 FAMEs (Supelco FAME37, Supelco 37-component mix, Supelco, Sigma Aldrich, St. Louis, MI, USA), along with individual standards purchased from Merck (Darmstadt, Germany) and Larodan (Solna, Sweden). Fatty acids were quantified by integrating chromatographic peak areas and applying internal-standard-based calculations, following the methodology described by Vahmani et al. [24]. Results were expressed as milligrams of fatty acid per 100 g of fresh meat.

### 2.5. Statistical Analysis

Data distributions were analyzed for normality assumptions before statistical testing. Comparisons between the fatty acid profiles of LD muscle and backfat within the BAP group were performed with a paired *t*-test using the software JMP Pro18 by SAS Institute (Cary, NC, USA).

To check if BAP samples were distinguishable from LD muscle from commercial pork, multivariate data analysis was used. The CP samples were employed only as a descriptive reference due to missing information about their origins. First, all quality parameters measured were merged into a 30 (samples) × 41 (variables) data matrix. Before multivariate analysis, data were standardized (scaled and mean-centered) and log10-transformed in order to avoid any biases related to data structure, namely removing the offset from the data, focusing on the biological variation and on the similarities (or dissimilarities) among the samples, and adjusting for the high fold differences observed between the variables. Principal Component Analysis (PCA) was first used as an unsupervised method to have a visual overview of any tendency of the samples from the two different groups (BAP and CP) to cluster in a reduced spatial dimension (bidimensional). After that, Partial Least Squares Regression Discriminant Analysis (PLS-DA) was used as a supervised method to maintain the dimensionality of the dataset reduction while trying to maximize the separation between the two groups under investigation, further analyzing the structure of the data obtained by laboratory analysis, including sample characteristics and variable significance. The classification was formulated as a binary problem with “BAP” as the positive class, encoding the response as y_01_ ∈ {0,1}. The number of LVs was selected based on the variance explained (SSQ table) and minimal cross-validation classification error. The first two latent variables (LV-1 and LV-2) were used for easy data interpretation. Predictions (continuous values 0–1) were converted into class labels using a 0.5 threshold. Model performance was evaluated using stratified 5-fold cross-validation (CV), preserving class proportions in each fold. For each fold, the PLS model was refitted on the training set, and predictions were generated for the test set. Aggregated CV metrics included accuracy, sensitivity, specificity, F1-score, balanced accuracy, and Matthews Correlation Coefficient (MCC). Statistical significance of all metrics (accuracy, balanced accuracy, MCC, AUC, Q^2^) was assessed via permutation testing (B = 1000 permutations) with randomly shuffled class labels, maintaining the CV framework.

Variable Importance in Projection (VIP) scores were used to investigate the most important variables for discriminating between BAP and LD samples in the multivariate dataset. Multivariate models were developed in the *mixOmics* package from RStudio (v 2025.09.1), while graphs were developed using the *ggplot2* package.

## 3. Results

### 3.1. Characterization of the Farming System Through On-Farm Data Collection

#### 3.1.1. Farming System

The herd composition of the farms involved in the study is presented in Table 2.

Herd sizes were small, ranging from 6 to 80 animals, a characteristic of traditional family farms breeding native breeds. All farms operate completely outdoors or with simple shelters. The spatial allocation across the surveyed outdoor pig farming operations revealed considerable variability in the amount of land per animal, ranging from 100 m^2^ per pig to more than 2500 m^2^ per pig. Most farms demonstrated substantial land availability per animal (>250 m^2^), falling short of the spatial requirements outlined in some regional guidelines for extensive farming [25], while two farms showed more limited spatial allocation, with densities around 100 m^2^ per pig. In all farms, the available space per animal significantly exceeded the minimum welfare requirements for conventional systems for weaner or rearing pigs, gilts, and sows [26]. In all farms, appropriate shelters were provided to meet the animals’ needs. In most farms, there are both adult sows and boars, with the presence of pigs raised in the fattening phase. The predominance of adult animals rather than growing stock in some farms (particularly Farm 6) reflects a production process primarily oriented toward reproduction, with the primary objective being the slaughter of piglets.

Housing management varied between farms. Four farms (F1, F3, F4, and F5) separated breeding adults from growing animals, while in one (F6), all animals were kept together, and one farm separated only the boars (F2). All farms reported using traditional paddock layouts rather than radial designs. During farrowing and lactation, all farms kept sows individually in dedicated structures with various dimensions (5–60 m^2^) and materials (sheet metal, wood, stone, fiberglass, or concrete). Environmental enrichment and protection were provided on all farms, including wooden feeding troughs and dedicated feeding stations, roofed areas for rain protection, trees, mud wallows, and plants. All farms combined natural feed resources with supplementary feeding. In fact, to address the uncertainty of feed availability due to climatic and seasonal variability, both conventional feed and a range of less commonly used local feed were supplemented to animals.

The natural vegetation varied from farm to farm. Most farms reported the presence of forest trees, such as oaks, chestnuts, beeches, cherries, locusts, and willows, which provided seasonal feed resources, particularly fallen acorns and chestnuts. Across the six farms, feeding strategies were highly diversified. The most common feed was grass (83%; 5/6 farms), followed by commercial feed (50%; 3/6), fruits, vegetables, and hay (each 33%; 2/6). A wide range of additional ingredients were sporadically used by single farms, including chestnuts, potatoes, cornmeal, corn grain, alfalfa, silage, topinambur, mixed cereals, whey, acorns, and dry bread (each 17%; 1/6). Water was provided through several systems, including water troughs and nipple drinkers.

#### 3.1.2. Productive and Reproductive Performance of BAPs

Average production parameters of BAPs are presented in Table 3.

The average daily gain (ADG) of the BAPs was about 150–200 g/day, reflecting a notably slow growth rate. The main production model in the farms is characterized by producing heavy pigs (140–180 kg live weight) at 24–48 months of age. The backfat thickness ranged from 40 to 80 mm, considerably higher than in modern lean breeds.

The reproductive data are influenced by the farm-specific management practices. The first farrowing for BAP sows occurs between 12 and 24 months (100–150 kg body weight), and the number of farrowings per sow per year is between one and two, with corresponding farrowing intervals of 300–365 days for farms reporting one farrowing per year and approximately 180–240 days for farms reporting two farrowings per year. Lactation periods were notably longer than in conventional systems, ranging from 60 to 120 days, allowing for more natural maternal behavior and piglet development. The litter size and characteristics are presented in Table 4.

Litter size ranged from 8 to 12 piglets born alive, with 1–3 stillborn piglets per litter. The number of weaned piglets (8–12) suggests relatively low pre-weaning mortality in most farms.

All farms had dedicated farrowing areas, and three farms additionally provided specific nests for piglets. None of the farms heated these nests, maintaining the natural behavior. The sows are reported to have excellent maternal behavior, tending to their offspring calmly and attentively and lying down carefully to avoid harming them.

Breeding was managed through natural mating on all farms. Two farms employed single-boar coverage, in which sows are moved after weaning to breeding areas where the boar is present; the sows remain with the boar for at least two weeks and then are transferred to the gestation paddock. One farm practiced controlled natural mating, in which a sow in estrus is served by the boar and subsequently returned to the sow group (while the boar returns to his enclosure); at each anticipated estrus cycle, the boar is introduced to identify receptive sows and perform remating as required. Two farms practiced uncontrolled natural mating. On half of the farms, boars and sows were kept together, while on the other half, they were managed separately, except during breeding.

### 3.2. Meat Quality

The pH, textural, and color parameters of the LD muscle samples of the BAPs are presented in Table 5.

**Figure 3:**
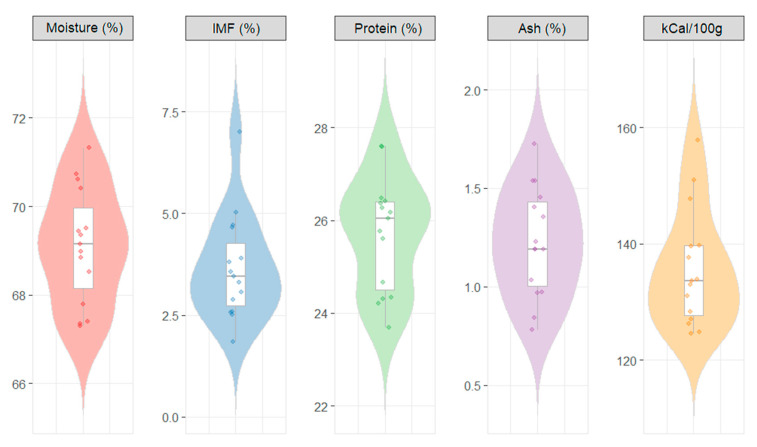
Violin and box plots for the proximate composition of LD muscle samples from BAPs.

The texture and color characteristics of the samples showed considerable variation across the measured parameters, as observable in Table 5. TPA showed that hardness had a mean value of 5.5 kg (SD = 1.3), with a median of 5.7 kg and an interquartile range of 1.2 kg. Adhesiveness displayed a mean of 3.6 g × s (SD = 2.3) and a median of 3.3 g × s. Springiness and cohesiveness both showed relatively low variability (CV = 0.1 and 0.0, respectively), indicating consistent values across samples. Chewiness presented a mean of 2.2 kg (SD = 0.7), while resilience demonstrated minimal variation with a mean of 0.3% (SD = 1.8). The WBSF test showed that firmness in BAP samples averaged 3.0 kg (SD = 0.5), and toughness reached a mean value of 12.1 kg × s (SD = 2.1). Regarding color parameters, L values averaged 45.3 (SD = 4.4), a* values showed a mean of 6.5 (SD = 2.1), and b* values averaged 11.0 (SD = 1.7). The pH measurements exhibited minimal variability (CV = 0.0), with a mean value of 5.4 (SD = 0.2). The proximate composition of LD muscle samples from the BAPs is presented in Figure 3.

The violin plots illustrate the distribution of proximate composition parameters and energy content across samples. Moisture content showed a relatively narrow distribution centered around 69%, with values ranging from 67% to 72%. Intramuscular fat (IMF) displayed a wider spread, with most values concentrated between 2.5% and 5.0%, and a median around 3.5%. Protein content exhibited a distribution centered near 26%, with values spanning approximately 24% to 28%. Ash content demonstrated considerable variation, with values distributed between 0.5% and 2.0%, with a median of around 1.2%. Energy content showed a distribution ranging from approximately 120 to 160 kCal/100 g, with the median value of around 135 kCal/100 g. The violin plots revealed that moisture and protein exhibited relatively symmetrical distributions, while IMF, ash, and energy content showed slightly skewed distributions with some outlying values visible as individual data points.

The fatty acid composition of loins from the BAPs is presented in Table 6.

The fatty acid composition of IMF in the BAP meat revealed distinct patterns across the major lipid classes (Table 6). Monounsaturated fatty acids (MUFAs) constituted the predominant group, with a mean content of 1672.9 mg/100 g of meat (representing 50.5% of total fatty acids), with oleic acid (18:1 n-9) being the most abundant individual fatty acid at 1340.2 mg/100 g (40.5%). Saturated fatty acids (SFAs) showed a mean of 1250.9 mg/100 g (37.8% of total fatty acids), primarily composed of palmitic acid (16:0; mean 787.2 mg/100 g, 23.8%) and stearic acid (18:0; mean 388.7 mg/100 g, 11.7%). Polyunsaturated fatty acids (PUFAs) averaged 378.0 mg/100 g (11.4% of total fatty acids), with linoleic acid (18:2 n-6; mean 261.6 mg/100 g, 7.9%) as the major component, followed by arachidonic acid (20:4 n-6; mean 69.0 mg/100 g, 2.1%), eicosadienoic acid (20:2 n-6; 11.7 mg/100 g; 0.3%), and α-linolenic acid (18:3 n-3; mean 11.1 mg/100 g; 0.3%). Among very long-chain PUFAs, EPA (20:5 n-3; mean 2.0 mg/100 g, 0.3%), DPA (22:5 n-3; mean 9.3 mg/100 g; 0.3%), and DHA (22:6 n-3; mean 1.9 mg/100 g; 0.1%) were also present, even if at very low concentrations. The omega-6-to-omega-3 ratio (n-6/n-3) exhibited considerable variation, with a mean of 14.2 (SD = 4.0) and a median of 14.3.

### 3.3. Fatty Acid Distribution in Meat and Backfat

The fatty acid composition of LD muscle and backfat (BF) from the BAPs is presented in Table 7.

A comparative analysis of FA distribution between IMF and BF in the BAP samples revealed some statistically significant differences between the two tissues. The total MUFA content was significantly lower in BF than in IMF. This trend was particularly pronounced for palmitoleic acid and vaccenic acid, whereas oleic acid exhibited an inverse pattern, being more abundant in BF than in IMF. No significant differences in total SFAs and PUFAs between the two anatomical portions were detected. Conversely, we observed significantly higher proportions of linoleic acid, α-linolenic acid, and eicosatrienoic acid (20:3 n-3) in BF, together with a lower concentration of arachidonic acid (20:4 n-6). Moreover, we found DHA (22:6 n-3) exclusively in the IMF of loins, even if just in trace concentrations (0.1%), while this FA was not detected in BF samples.

### 3.4. Multivariate Fingerprint of BAP Meat

**Figure 4:**
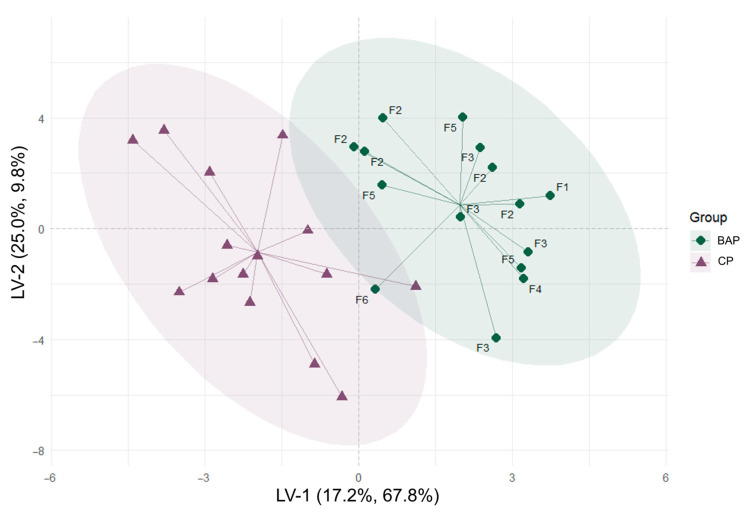
PLS-DA score plot. F1, F2, F3, F4, F5, F6 = Farm number.

In the PCA analysis, PC-1 and PC-2 accounted for 44.2% of the total variance in the original data matrix. This plot showed a slight tendency to cluster BAP and LD samples over the direction of PC-2, but high overlapping between the two groups (17 out of 30 samples) was observed (Supplementary Figure S1). For this reason, PLS-DA was performed to evaluate relationships between samples and identify patterns in the physicochemical characteristics and fatty acid composition of the BAP and LD meat samples. A PLS-DA model was built through a Venetian Blind Cross-Validation method with 10 data splits, one sample per split, and initially retaining a total of 20 latent variables (LVs). The evaluation of the cross-validation results allowed us to build a discrimination model using two LVs, retaining 32.9% of cumulative variance on X and 77.6% total variance on Y, with accuracy = 0.83, sensitivity = 0.80, specificity = 0.87, MCC = 0.67, and AUC = 0.92, for a total of 25 out of 30 samples correctly classified (classification error = 0.167). The results from the permutation tests performed on these metrics are available in Supplementary Materials Figure S2. The overlap between BAPs and LDPs in the PLS-DA score plot (Figure 4) was minimal, with one BAP (from F6) sample clustering in the intersection between the 95% confidence ellipses of the two groups, and vice versa. In the PLS-DA score plot (Figure 4), the discrimination of the centroids of the two groups occurred in the direction of LV-1.

**Figure 5:**
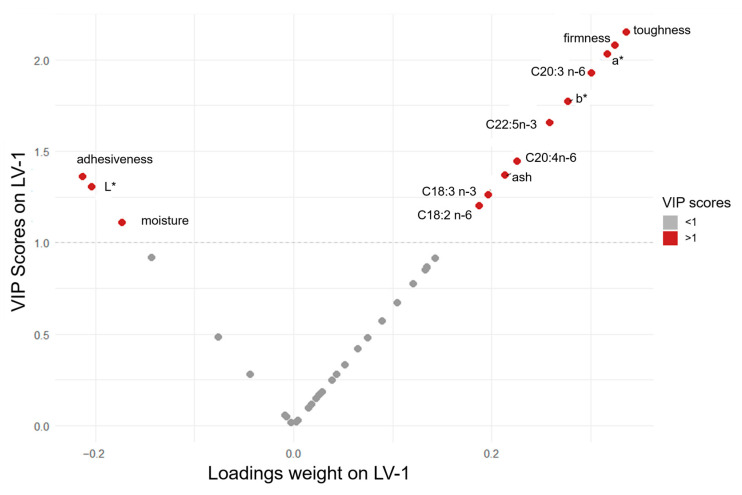
VIPs (on the Y-axis) and loading weights (on the X-axis) of original variables for the first latent variable (LV-1) calculated by the PLS-DA model. Variables in red are those with the highest loading weight (both positive and negative values) and characterized by VIP scores > 1.

The first latent variable was the principal driver of the separation between the two groups, while the second one contributed only marginally. The analysis of the score plot showed that the BAP meat samples clearly clustered on the right side of the plot (positive values on LV-1), while the LD samples were grouped on the left side (negative values on LV-1). Loadings and VIP (Variable Importance in Projection) scores on LV-1 are presented in Figure 5.

**Figure 6:**
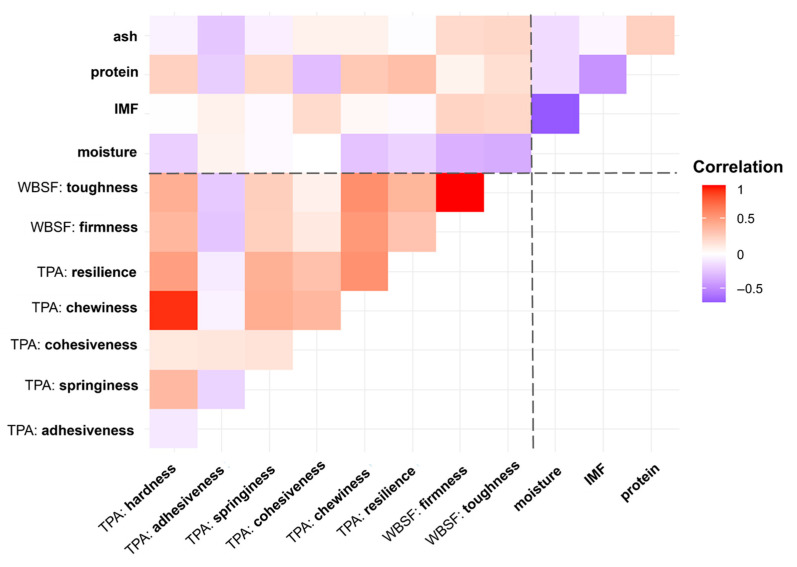
Correlation matrix heatmap between textural and chemical features of BAP loin samples.

The VIP values allowed us to link the good discrimination between the centroids of the two groups, BAP and LD, to specific quality features analyzed in this study. The variables associated with the highest VIP values are those associated with the color of the sample (a*, b*, and L*), three textural quality traits (toughness, firmness, and adhesiveness), and ash and moisture contents, plus a group of PUFAs of the n-3 and n-6 series. No significant correlations were detected between texture parameters and the chemical composition of the meat (Figure 6), and no relationship was observed between IMF content and meat tenderness.

## 4. Discussion

In this exploratory study, the phenotypical traits and the farming systems employed at present on BAP farms are presented for the first time. The characteristics typical of the farming systems employed on the BAP farms investigated in this study evidenced the presence of the conditions necessary to define the BAP production system as “en plein air”. Considered an evolution of traditional extensive farming, this rearing system involves housing animals in fenced outdoor areas equipped with water sources, shelters, and supplemental feeding systems [17].

Based on our results, the BAP can be defined as a slow-growing genetic type with high mobility and natural foraging abilities. The BAP is currently undergoing recovery, raised in small farming groups with highly variable feeding systems often based on available on-farm resources such as vegetables, grains, and fruits. This heterogeneous management contributes to their slower and variable growth performance relative to both commercial hybrids and better-optimized native breeds. The limited use of commercial feed and ad libitum intake of roughage results in reduced energy intake and average daily gain in growing pigs [27].

This is particularly evident when compared to commercial hybrids and is related to the different production systems (intensive vs. extensive) and highly heterogeneous feeding regimes of BAPs. Commercial hybrids destined for dry-cured ham production typically achieve ADG values between 670 and 690 g/day [28], and even higher rates (921–966 g/day) are recorded in lighter pigs raised for fresh meat [29]. BAPs also grow more slowly than other native Italian breeds, which have benefited from recent optimization in farming practices and feeding strategies. For example, the Cinta Senese reaches 393–443 g/day [30], Casertana pigs and Mora Romagnola fed commercial diets exhibit an ADG of approximately 376–415 g/day, and Nero Siciliano pigs grow from 77 g/day in extensive systems to 320 g/day under en plein air management [17,31].

Backfat thickness in BAPs ranged from 40 to 80 mm, markedly exceeding the values typically observed in modern lean breeds and reinforcing the traditional characteristics of these production systems. For comparison, commercial hybrids, reared for dry-cured ham production and slaughtered at about 9 months of age, show backfat thickness of 22.5–24.8 mm at 145 kg [29] and 30–32 mm at 170–180 kg [28]. Among other native Italian breeds, Cinta Senese pigs display 28.7–30.3 mm backfat thickness [30]; Casertana and Mora Romagnola fed commercial diets show even greater values—48 mm and 57 mm, respectively [31]—while Nero Siciliano pigs range from 34 to 47 mm [17]. These higher levels of backfat in traditional breeds are consistent with their adaptation to extensive systems, where increased fat reserves play a key role in thermoregulation and help to compensate for seasonal feed shortages or low-quality diets. As noted by Edwards et al. [32], such breeds typically develop thicker and firmer fat layers as a physiological response to these challenging conditions. Even the reproductive characteristics of the BAP showed lower reproductive efficiency than the intensive systems, but they align with the seasonal nature of traditional outdoor pig production.

The adoption of extensive and semi-extensive farming systems in pig farming leads to substantially improved quality traits in meat [9]. It must be specified that, given the marked heterogeneity among the six farms, the meat quality traits observed in BAPs were expected to reflect not only genetic characteristics but also farm-specific environmental and managerial influences. Because feeding regimes, housing conditions, sex, reproductive management, and slaughter age/weight differed among farms, these differences should be interpreted as representative of the current production contexts in which the BAP is raised, rather than as strictly comparable outputs from standardized systems. We believe that this variability does not weaken the study; rather, it underscores the real-world conditions of this local breed and defines the boundaries within which the current production context of the BAP can be interpreted, thereby providing an authentic overview of its performance across different alpine and lowland farming environments. In this respect, our observations should be viewed as contextual rather than prescriptive: they delineate the performance envelope of the BAP under the diversity of practices presently adopted by farmers engaged in conserving this breed. These results are particularly valuable at this early stage of recovery, as they highlight both the potential of the breed and its management’s variability, which future initiatives may gradually reduce.

The L*, a*, and b* values detected in BAP LD muscle were comparable to those reported by other authors for some native and rustic breeds slaughtered at similar body weights (145–174 kg) [33,34,35]. In the literature, an enhanced redness in pork meat has been attributed to elevated myoglobin concentrations, which are likely associated with a predominantly oxidative metabolism of animals reared outdoors, promoted by increased physical activity, and to a substantially extended slaughter age [9]. The high yellowness in BAP LD muscle may be related to outdoor rearing through the incorporation of some compounds derived from dietary plant sources (grass), which impart a yellow (lutein) and orange (beta-carotene) pigmentation to adipose tissue in animal-derived food products [36]. It can be argued that BAP meat, from slow-growing pigs reared in outdoor farming systems and with access to green forage, contains higher levels of carotenoids and lutein in fat than confined pigs. This has been observed in other animal species, including pork, grazing ruminants, and outdoor-reared poultry [37,38].

The WBSF analysis showed values for firmness and tenderness comparable to those of other local genetic lines reared in unconventional farming systems [33,34]. As pointed out by Pugliese and Sirtori [9], meat from pigs that have been reared outdoors and slaughtered at an older age is generally firmer and tougher than meat from commercial hybrids. Following the classification of pork meat in tenderness categories and the association with WBSF according to the scores given by the sensory panel reported in Van Oeckel et al. [39], 7 out of 15 BAP LD muscles analyzed could be defined as “tender” (WBSF ≤ 3.0 kg), 6 out of 15 as “intermediate” (3.0 kg < WBSF < 3.6 kg), and 2 out of 15 as “tough” (WBSF > 3.6 kg). Many authors have suggested that TPA may provide a more accurate prediction of meat’s sensory tenderness compared to the WBSF test, whether in beef [40] or pork [41]. This stems from TPA’s ability to better simulate the multifaceted chewing process, whereas WBSF only evaluates resistance during a single slice. Unfortunately, there is a lack of studies reporting TPA results for native pork meat. Overall, we found higher values for hardness and cohesiveness (along with lower values for springiness, adhesiveness, chewiness, and resilience) than those found in loins from commercial hybrid genetic lines, reared and slaughtered under common commercial conditions [42].

Comparing the proximate composition of LD muscle from BAPs and published data on cosmopolitan pig genetics, the results are conflicting due to the different genetic types, feeding regimes, and nutritional characteristics of the diet. Compared to commercial hybrids fed ad libitum and slaughtered at about 126 kg of live weight and at about 167 days, lower intramuscular fat (IMF) and higher protein contents have been observed in BAPs on average [43]. Another study reported that in medium–heavy pigs, with restricted feed regimens (slaughtered at 130 kg of live weight), the LD muscle IMF ranged from 3.1% to 3.5% [44]. In heavy pigs, slaughtered at 166 kg of live weight, with a restricted feed regimen, the IMF of the LD muscle is about 2.5% [45]. On the other hand, the average IMF content observed in BAP LD muscle (3.7%) aligned with values reported for other Italian native black pig breeds reared both indoors and outdoors, which ranged from 2% to 5% IMF [9,46]. The IMF content in BAP loins is above 2.5–3%, which is generally considered the minimum threshold to achieve acceptable palatability in terms of juiciness and flavor in fresh pork [47].

The fatty acid profile detected in the BAPs reflected the characteristic lipid composition of pork IMF in medium and heavy pigs, from both commercial hybrids intensively raised for ham production [48] to local genetic lines reared *en plein air*, such as Nero Siciliano pigs [17]. However, the variability in the FA values for BAP meat analyzed in this study (Table 6) highlighted substantial variability in fatty acid composition among the samples, likely consistent with heterogeneity in farm management and feeding practices.

Even if the sampling consistency does not allow us to identify any statistically supported difference among the farms, it can be observed that the overall BAP exhibited lower oleic acid content (1.3 g/100 g of fresh meat) compared to several rustic pig breeds reported in the literature, which typically receive diets rich in oleic acid through specialized feed formulations or traditional acorn and chestnut feeding [9]. Relatedly, high total unsaturated fatty acid content, particularly PUFAs (378.0 mg/100 g of meat, on average), was observed. This distinctive feature could stem from the outdoor rearing system implemented for BAPs on all the farms, allowing for continuous access to fresh grass and natural vegetation (including garden scraps), which are abundant sources of essential PUFAs. This outcome is very interesting, considering the functional and nutritional implications of PUFAs, particularly those belonging to the n-3 series. Very long-chain n-3 PUFAs, such as EPA and DHA, contribute to cardiovascular protection, reduce inflammation, and support neural development. Increasing the n-3 PUFAs in pork meat, thus lowering the n-6/n-3 ratio, aligns with dietary recommendations for reducing chronic disease risk. Thus, pork rich in n-3 PUFAs represents a valuable source of health-promoting fatty acids in human diets without requiring major changes in eating habits [49]. Grass, particularly enriched in α-linolenic acid (18:3 n-3), comprising up to 50% of total fatty acids in plants [50,51], contributes to the distinctive fatty acid profile observed in outdoor-raised pigs, which show high levels of PUFAs and n-3 fatty acids in muscle tissue compared to indoor-raised pigs [32]. Our findings are consistent with those recently reported by Ianni et al. [46], who documented higher α-linolenic acid levels in IMF from outdoor-raised black pigs compared to commercial hybrids.

In comparison with Casertana pigs slaughtered at 370 days of age, 155 kg live weight, and BAP LD muscle contained lower proportions of SFAs and PUFAs but higher MUFAs [52]. Moreover, the elevated linoleic acid content in Casertana meat resulted in a substantially higher n-6–n-3 ratio of 31.9 (calculated as linoleic + arachidonic/α-linolenic), approximately twice the value observed in BAPs. This is probably due to the fact that, in the cited study, even if reared outdoors, Casertana pigs were fed a commercial corn-based diet.

The BAP revealed striking differences in fatty acid composition, even compared to Iberian pigs (IPs), containing higher PUFAs and lower MUFAs. This divergence can be attributed to differences in traditional feeding practices, with Iberian pigs typically consuming large quantities of acorns during the Montanera period [51], resulting in exceptionally high oleic acid incorporation into their muscles. In contrast, BAPs, raised outdoors in areas where grass is seasonally available, probably accumulated more PUFAs through consumption of fresh grass (rich in α-linolenic acid) and other naturally available vegetation. Compared to free-range fattened IPs slaughtered at 170–175 kg [35], the BAPs contained higher PUFAs, lower MUFAs, and comparable SFAs. Notably, the Iberian pigs in the referenced study were maintained under controlled rearing conditions from birth through the fattening phase and received diets including acorns and grass (free-range) or maize, alfalfa meal, wheat, and high-oleic-acid sunflower oil (experimental diets).

Comparison of the fatty acid composition of the two anatomical portions investigated (LD and BF) revealed results consistent with the findings of Sun et al. [53] for loin and backfat samples from a broad range of pig genotypes. Their study similarly reported significant differences in FA composition between these anatomical regions, including lower overall MUFA content and reduced levels of palmitic acid in BF compared to IMF.

This differential distribution of FAs likely reflects the distinct lipid compositions of the two tissue types. The *L. dorsi* muscle features an extensive network of cellular membranes that necessitate a specific phospholipid composition, with a preferential incorporation of arachidonic acid due to its essential roles in maintaining membrane fluidity and mediating cell signaling pathways. In contrast, adipose tissue primarily consists of triglycerides, with linoleic acid commonly present in its unmodified form, serving mainly as an energy reserve. Linoleic and linolenic acids are higher in pig adipose tissue than in muscle, while the latter contains significant proportions of long-chain unsaturated FAs (20–22 carbon atoms) formed by elongation and desaturation of linoleic and α-linolenic acids [54]. Notably, given the limited α-linolenic acid content in the BF samples analyzed, the corresponding elongation and desaturation derivatives were barely detectable (<0.5%). In contrast, the abundant linoleic acid substrate resulted in clearly distinguishable tissue-specific differences between IMF and BF, most notably in arachidonic acid concentrations, the main product of linoleic acid elongation and desaturation pathways.

Pugliese and Sirtori [9] reported that indigenous pig breeds exhibit a marked propensity for MUFA accumulation, particularly oleic acid, in contrast to commercially selected breeds, which tend to show higher concentrations of SFAs or linoleic acid. According to the authors, this distinctive FA profile in heritage breeds likely arises from inherent differences in de novo lipogenesis pathways, metabolic turnover rates, and a greater capacity to incorporate MUFAs into tissue—an ability that becomes more pronounced with age. In line with these observations, our analysis revealed higher MUFA concentrations in the BF of BAPs compared to values reported for heavy commercial hybrid pigs optimized for rapid growth and dry-cured ham production, along with elevated levels of oleic acid [48]. This enrichment in MUFAs has dual implications: nutritionally, it improves fat quality by reducing saturated fat intake and increasing oleic acid, which is associated with cardiovascular benefits; technologically, a higher proportion of MUFAs lowers the fat melting point, making backfat softer and less firm, which can affect processing and storage stability. Moreover, increased unsaturation raises susceptibility to oxidation, potentially impacting the shelf life and sensory attributes of cured products [55].

Furthermore, the BAP BF samples showed lower SFA levels and higher concentrations of both linoleic and linolenic acids compared to the commercial hybrids heavy pigs included in the study by Comin et al. [48]. These differences are likely related to the metabolic characteristics underlying adipose tissue development in the swine species, with elevated oleic acid content in BF reflecting the central role of this fatty acid in endogenous fat synthesis within adipose tissue [35].

The multivariate data analysis approach was used in this study to determine the positioning of BAP LD muscle relative to the CP samples, considered exclusively as a descriptive reference. We acknowledge that this comparison does not support inferential or causal interpretation. The evaluation of the results from the multivariate data analysis showed that some individual features related to meat quality (VIPs) had higher importance in separating the BAP meat from the CP meat. These traits likely stem partially from the genetic background and mostly from the traditional rearing practices associated with BAPs, resulting in meat with specific textural and compositional attributes.

The distribution of scores in the PLS-DA plot suggested meat darker with more intense red–yellow components, along with higher toughness, higher firmness, less water content, and higher ash for BAP. Color feature incidence in discriminating the two groups could be potentially due to differences in myoglobin content and other compounds responsible for the red and yellow color in BAP meat, as commonly observed in meat from pigs reared in an outdoor farming system with access to natural resources. Further studies would be necessary to clarify the reasons for this difference. However, since color appeared as a key trait in discriminating between the BAP and LD samples, ΔE_Lab_ was calculated as the difference between the two colors [56], as follows:


ΔE_Lab_ = √ [(ΔL*)^2^+ (Δa*)^2^+ (Δb*)^2^)] (1)

As a result, the ΔE_Lab_ observed between the color detected for BAP and CP in the present study was equal to 5.6, which is the range considered easily noticeable to the human eye (ΔE_Lab_ > 5) and, consequently, appreciable to consumers. This is very important since color is one of the fundamental aspects of quality considered by consumers in their purchasing decisions for meat. Meanwhile, the higher hardness and toughness values observed in BAP meat, suggesting a potentially firmer texture, are more likely associated with differences in age at slaughter or quantitative differences in connective tissue.

Regarding fatty acid composition, the analysis of the loadings and VIP scores revealed that BAP loins were associated with high concentrations of linoleic, α-linolenic acid, eicosatrienoic acid, arachidonic acid, and DPA. Even if some differences in the BAP are attributable to the farm of origin, our findings support the literature demonstrating that pigs with access to pastures typically display elevated levels of PUFAs (including the n-3 series) in muscle tissue compared to indoor-raised pigs.

Contrary to our expectations, when investigating the correlation matrix between the chemical and textural features in meat, our results showed no significant correlation between IMF content and meat tenderness. One study by Rincker et al. [57] reported that WBSF and extractable lipid content in pork were negatively correlated with a very weak relationship (R^2^ = 0.10), and even the results of consumer tests showed that intramuscular fat content had limited effects on perceived tenderness. Although IMF is known to improve tenderness by disrupting connective tissue structure and weakening the overall structural integrity of meat, it has been reported that this effect becomes significant primarily in heavily marbled muscles. In fact, IMF can influence pork tenderness and texture, and its effect tends to become perceptible only when IMF content exceeds 2% [47]. Conversely, IMF values lower than 2% can decrease meat tenderness and sensory quality [58]. This threshold is substantially lower than the average IMF value (3.7%) recorded in our study. Recently, Li et al. [42] reported negative correlations between IMF content and WBSF values in pig loins from different genetic lines. Notably, despite reporting correlations between IMF content and WBSF values, the authors found no significant associations between IMF and parameters from TPA, which is consistent with our findings.

## 5. Conclusions

This exploratory study provides the first comprehensive characterization of the Black Alpine Pig farming system and related meat quality traits. The presented data portray the BAP as a rustic breed managed in heterogeneous outdoor systems, producing meat with distinctive characteristics shaped by the environmental context and managerial conditions of each farm. By documenting this little-known but valuable genetic resource, our study offers a descriptive overview of BAP meat quality traits and contributes to the current efforts in biodiversity conservation, supporting the alpine livestock farming system and the recovery of local rural areas.

These findings provide important data to identify which traits should be preserved, improved, or promoted to support the conservation and valorization of the breed, its farming system, and its products in the local market, along with potential strategies for improvement.

Consistent with the exploratory nature of this study, our results also highlighted some detectable meat quality differences between outdoor-raised BAPs and CPs, while similarities with other black pigs were confirmed. Future research should include larger samples and standardized management practices and explore how environmental, genetic, and dietary factors may influence meat quality to improve product characteristics without compromising sustainability and authenticity.

## Figures and Tables

**Table 1:** Location, altitude, and size of the farms.

	Farm 1	Farm 2	Farm 3	Farm4	Farm 5	Farm 6
Province	Aosta	Sondrio	Alessandria	Bolzano	Sondrio	Como
Region	Valle d’Aosta	Lombardia	Piemonte	Alto Adige	Lombardia	Lombardia
Altitude (*m asl*)	1000	450	200	1500	1200	400
Farm size (*ha*)	0.9	12	30	6	4	0.6
Total animals	20	30	15	9	78	6

**Table 2:** Herd composition of BAP farms.

	F1	F2	F3	F4	F5	F6
N° of sows	2	2	2	1	5	5
N° of gilts/young boars	1	2	0	8	0	0
N° of boars	0	1	1	1	2	1
N° of fattening pigs	18	25	12	0	71	0
Total animals	21	30	15	10	78	6

**Table 3:** Average production parameters on BAP farms.

Production Parameter	Average Value
Live weight at slaughter (*kg*)	140–180
Age at slaughter (*months*)	24–48
Average daily gain (*g per day*)	150–200
Carcass weight (*kg*)	115–150
Carcass yield (%)	82–83
Backfat thickness (*mm*)	40–80

**Table 4:** Litter size and characteristics on BAP farms.

Parameter	Average Value
Piglets born alive per litter	8–12
Stillborn piglets per litter	1–3
Weaned piglets per litter	8–12
Piglet birth weight (*g*)	800–1000

**Table 5:** Textural parameters, color, and pH of *Longissimus dorsi* muscle (N = 15) from Nero delle Alpi (BAP) pigs divided by farm of origin. Data are presented as mean (min; max), standard deviation (SD), median (Q1; Q3), interquartile range (IQR), and coefficient of variation (CV).

Variable	Mean (min; max)	SD	Median (Q1; Q3)	IQR	CV
*Texture*					
Hardness (*kg*)	5.5 (2.7; 8.5)	1.3	5.7 (4.7; 5.9)	1.2	0.2
Adhesiveness (*g* × *s*)	3.6 (0.6; 8.5)	2.3	3.3 (2.3; 3.9)	1.7	0.6
Springiness (%)	0.6 (0.5; 0.7)	4.4	0.6 (0.6; 0.6)	0.0	0.1
Cohesiveness (%)	0.6 (0.6; 0.7)	2.5	0.6 (0.6; 0.6)	0.0	0.0
Chewiness (*kg*)	2.2 (0.9; 3.5)	0.7	2.2 (1.7; 2.7)	1.0	0.3
Resilience (%)	0.3 (0.2; 0.3)	1.8	0.3 (0.2; 0.3)	0.0	0.1
Firmness (*kg*)	3.0 (2.0; 4.0)	0.5	3.1 (2.9; 3.1)	0.3	0.2
Toughness (*kg* × *s*)	12.1 (7.3; 15.3)	2.1	12.5 (12.2; 13.2)	1.0	0.2
*Color*					
L*	45.3 (37.6; 53.0)	4.4	45.1 (43.4; 47.7)	4.3	0.1
a*	6.5 (2.9; 11.0)	2.1	5.9 (5.6; 7.2)	1.6	0.3
b*	11.0 (7.8; 13.9)	1.7	10.5 (9.8; 12.5)	2.7	0.2
pH	5.4 (5.0; 5.8)	0.2	5.5 (5.2; 5.7)	0.5	0.0

**Table 6:** Fatty acid profile of *Longissimus dorsi* muscle (N = 15) from Nero delle Alpi (BAP) pigs divided per farm of origin. Data are presented as mg of fatty acid/100 mg of total fatty acids. Data are presented as mean (min; max), standard deviation (SD), median (Q1; Q3), interquartile range (IQR), and coefficient of variation (CV).

Fatty Acid	Mean (min; max)	SD	Median (Q1; Q3)	IQR	CV
12:0	3.2 (1.4; 7.4)	1.9	2.3 (1.9; 4.1)	2.2	61.2
14:0	47.5 (20.5; 104.5)	26.3	37.9 (30.6; 56.8)	26.3	55.5
15:0	1.4 (1.0; 2.5)	0.4	1.3 (1.1; 1.5)	0.4	27.0
16:0	787.2 (383.5; 1571.7)	381.6	633.3 (513.7; 936.2)	422.6	48.5
17:0	6.6 (4.4; 10.8)	1.9	6.4 (5.0; 7.2)	2.2	28.6
18:0	388.7 (206.2; 816.6)	191.2	342.4 (240.0; 467.4)	227.4	49.2
20:0	7.3 (3.2; 17.4)	3.7	6.2 (4.8; 8.4)	3.5	51.1
22:0	1.4 (0.6; 2.7)	0.7	1.1 (1.0; 1.8)	0.8	51.5
24:0	7.7 (5.4; 12.9)	2.1	7.1 (6.0; 8.9)	2.9	27.9
∑SFAs	1250.9 (646.8; 2515.5)	604.6	1034.3 (807.4; 1480.8)	673.4	48.3
14:1	0.9 (0.4; 1.8)	0.4	0.7 (0.6; 1.0)	0.4	46.9
16:1	122.9 (54.9; 246.3)	51.8	104.1 (92.8; 138.9)	46.1	42.1
18:1 n-9	1340.2 (583.8; 2564.0)	600.3	1144.9 (907.7; 1611.7)	704.0	44.8
18:1 n-7	175.0 (89.3; 307.6)	59.8	163.4 (135.6; 198.3)	62.7	34.2
C20:1 n-9	32.9 (15.8; 54.1)	13.0	29.6 (22.2; 41.5)	19.3	39.4
C24:1 n-9	1.0 (0.6; 1.7)	0.3	0.9 (0.8; 1.1)	0.3	31.6
∑MUFAs	1672.9 (745.5; 3175.4)	721.2	1429.0 (1154.4; 1986.4)	832.0	43.1
18:2 n-6	261.6 (194.6; 405.9)	63.9	235.5 (213.1; 285.4)	72.2	24.4
18:3 n-6	1.3 (0.9; 2.1)	0.3	1.3 (1.1; 1.5)	0.4	24.2
18:3 n-3	11.1 (5.1; 21.5)	4.9	10.5 (7.2; 12.7)	5.5	43.7
20:2 n-6	11.7 (6.6; 20.6)	4.2	11.0 (8.7; 13.5)	4.9	35.4
20:3 n-6	8.4 (6.3; 12.0)	1.5	8.0 (7.4; 9.4)	2.1	18.3
20:3 n-3	3.4 (0.7; 5.9)	1.5	3.3 (2.4; 4.2)	1.9	44.6
20:4 n-6	69.0 (49.4; 92.2)	11.4	70.0 (63.5; 75.2)	11.8	16.5
20:5 n-3	2.0 (0.7; 4.8)	1.0	1.9 (1.5; 2.1)	0.6	50.8
22:5 n-3	9.3 (5.5; 12.1)	2.1	9.6 (7.7; 11.3)	3.5	23.0
22:6 n-3	1.9 (0.8; 3.1)	0.7	1.5 (1.3; 2.4)	1.1	37.5
∑PUFAs	378.0 (289.4; 547.2)	73.8	369.6 (321.7; 402.2)	80.5	19.5
n-3	27.7 (19.2; 41.7)	6.9	27.6 (22.1; 30.9)	8.8	24.8
n-6	380.4 (274.7; 607.0)	106.6	349.7 (306.2; 393.8)	87.6	28.0
n-6/n-3	14.2 (7.9; 19.7)	4.0	14.3 (10.8; 18.6)	7.8	28.3

**Table 7:** Fatty acid composition of IMF (n = 15) and backfat (BF, n = 15) from BAPs and statistical significance (paired *t*-test). FAs are expressed as mg of FA/100 mg of total identified FAs, mean ± standard deviation. Only FAs detected at percentages > 0.1% are presented in the table.

	IMF n = 15	BF n = 15	*p*-Value
14:0	1.4 ± 0.2	1.5 ± 0.2	*ns*
16:0	23.3 ± 1.7	23.5 ± 2.3	*ns*
17:0	0.2 ± 0.1	0.4 ± 0.1	<0.001
18:0	11.6 ± 1.4	11.9 ± 1.4	*ns*
20:0	0.2 ± 0.1	0.2 ± 0.1	*ns*
24:0	0.3 ± 0.1	0.1 ± 0.0	<0.001
ΣSFAs	37.2 ± 2.9	37.8 ± 3.5	*ns*
16:1	3.7 ± 0.5	2.4 ± 0.4	<0.001
18:1 n-9	40.0 ± 2.3	43.0 ± 1.7	0.003
18:1 n-7	5.4 ± 0.8	3.5 ± 0.4	<0.001
20:1 n-9	1.0 ± 0.2	nd	<0.001
ΣMUFAs	50.3 ± 2.8	48.5 ± 2.2	*ns*
18:2 n-6	8.6 ± 2.2	12.0 ± 3.1	<0.001
18:3 n-3	0.4 ± 0.1	0.7 ± 0.3	<0.001
20:2 n-6	0.4 ± 0.1	0.7 ± 0.2	<0.001
20:3 n-6	0.3 ± 0.1	0.1 ± 0.0	<0.001
20:3 n-3	0.1 ± 0.1	0.2 ± 0.1	0.039
20:4 n-6 (*ARA*)	2.4 ± 1.0	0.1 ± 0.1	<0.001
22:5 n-3 (*DPA*)	0.3 ± 0.2	0.1 ± 0.0	<0.001
ΣPUFAs	12.6 ± 3.5	13.9 ± 3.6	*ns*
Σn-3	0.9 ± 0.3	0.9 ± 0.4	*ns*
Σn-6	13.4 ± 6.3	12.9 ± 3.3	*ns*
n-6/n-3 ratio	14.2 ± 4.0	14.9 ± 5.3	*ns*

*ns* = not significant.

## Data Availability

Due to administrative and legal procedures, raw data from this research cannot be shared at this stage.

## Supplementary Materials

The following supporting information can be downloaded at https://www.mdpi.com/article/10.3390/ani16010022/s1: Figure S1: PCA scores plot. Figure S2: Plots of the permutation test (B = 1000) for Accuracy, Sensitivity, Specificity, MCC and AUC obtained by the PLS-DA model with CV.
