# Supplementary material for: An Exploratory Study on Farming System and Meat Quality of Black Alpine Pig
Source: Animals (Basel). 2025 Dec 21;16(1):22. doi: 10.3390/ani16010022 (PMC12784736; doi:10.3390/ani16010022)

Supplementary materials Figure S1. PCA scores plot.

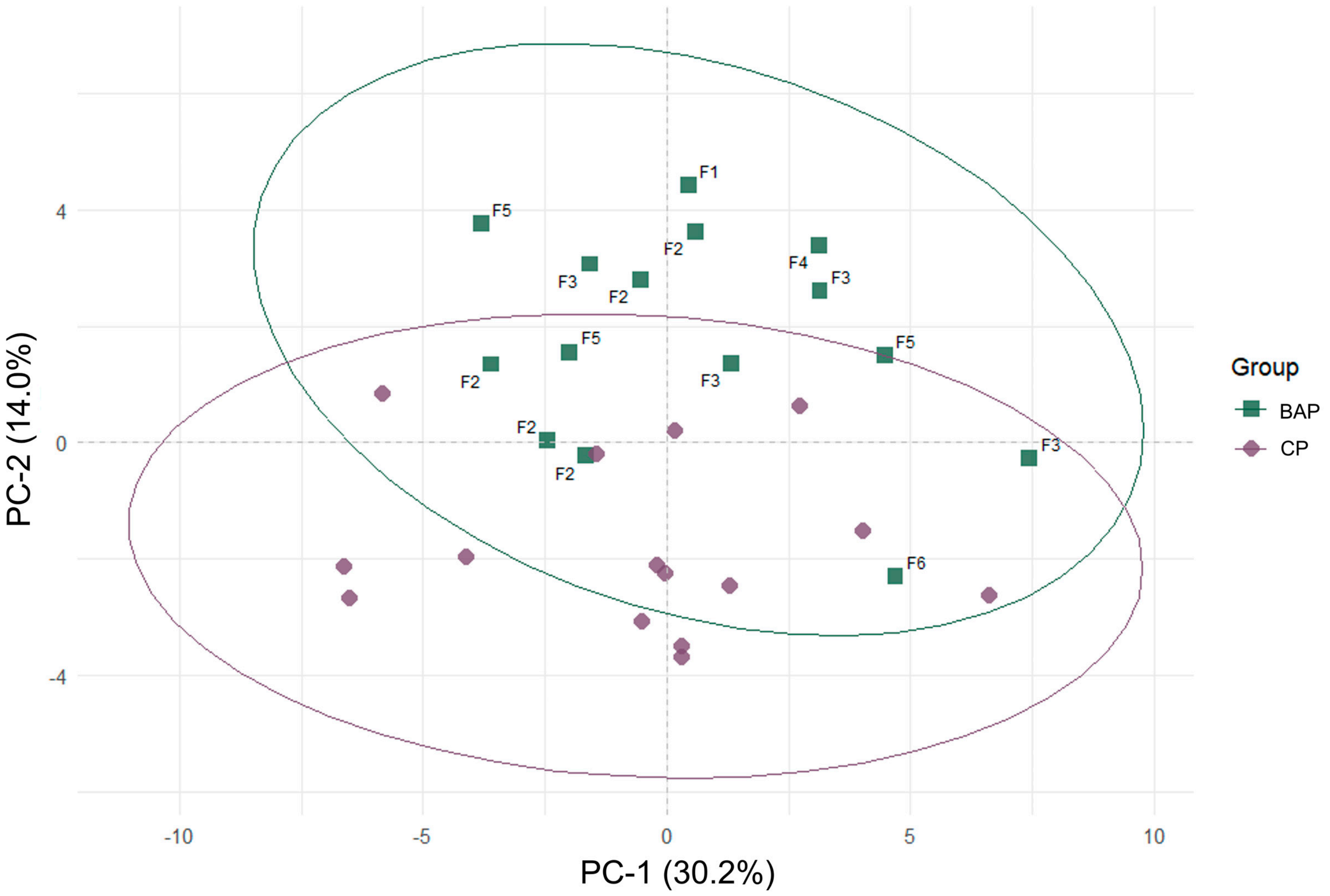

Supplementary Material Figure S2. Plots of the permutation test (B=1000) for Accuracy, Sensitivity, Specificity, MCC and AUC obtained by the PLS-DA model with CV.

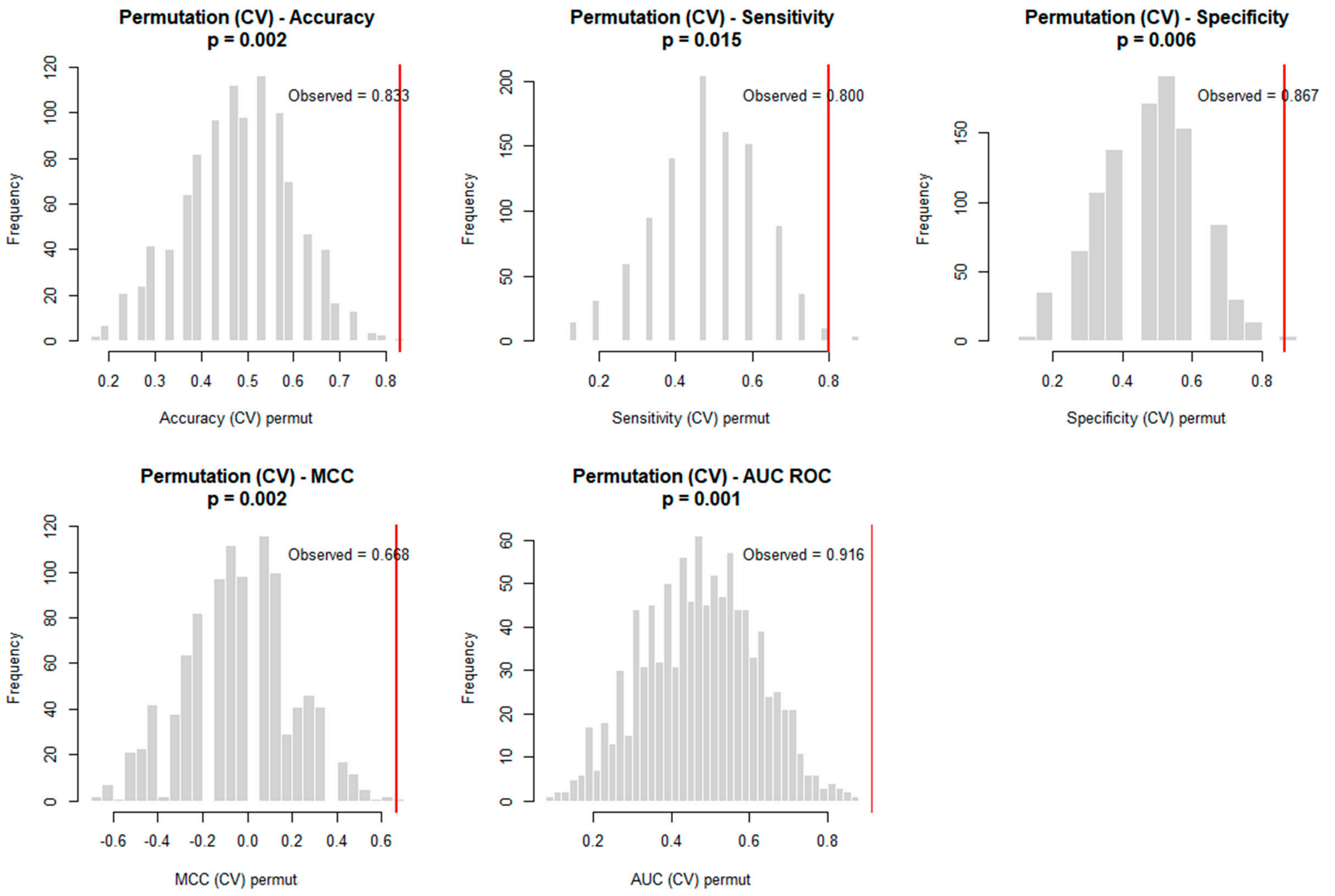

Supplement: Supplementary file 1 [file animals-16-00022-s001.zip › animals-4037341-supplementary.pdf]
